# Medical Ethics and the Biopsychosocial Model for Patient Care: A Case Analysis for Improved Communication, Clinical Time, and Error Avoidance

**DOI:** 10.7759/cureus.8535

**Published:** 2020-06-09

**Authors:** Dharam Persaud-Sharma, Marien Govea, Robert Hernandez

**Affiliations:** 1 Internal Medicine, Kendall Regional Medical Center, Herbert Wertheim College of Medicine Florida International University, Miami, USA; 2 Internal Medicine, Florida International University, Herbert Wertheim College of Medicine, Miami, USA; 3 Internal Medicine/Infectious Disease, Kendall Regional Medical Center, Miami, USA

**Keywords:** biopsychosocial model, bioethics, clinical care, cultural medicine, communication

## Abstract

The practice of interdisciplinary medicine is one of the most effective and cooperative forms of medical management, which optimizes clinical care and outcomes for a patient. This model of care affords the patient the benefit of receiving the best available therapeutic options from specialists who are experts in their respective disciplines, which would otherwise be limited when compared with the clinical expertise from a single provider managing multiple co-morbidities. However, poor communication between each specialized team managing a patient's care can result in redundancies and superfluous treatment that can have deleterious clinical outcomes that impede the physician-patient relationship and question the bioethical principles of clinical practice. Having a medical provider like an internist who is the primary medical provider for a patient anchors reinforces the physician-patient relationship through familiarity and continuous involvement in the gross clinical course of a patient. Specialty care provides a very focused and limiting scope of practice. However, whether practicing specialty care or being a generalist, utilizing clinical tools, such as the biopsychosocial model and routinely using bioethical principles during clinical encounters, not only help extract pertinent information from the patient's medical history but also furthers the continuity of clinical care by understanding the global context of the patient's medical history. This is a case analysis that exemplifies sub-optimal outcomes in patient care due to undermining the critical role of an internist in patient care and clinical management in addition to challenging several bioethical principles of clinical care. It also highlights the importance of how using the biopsychosocial model of care can avoid clinical errors, improve interdisciplinary and patient communication, and, ultimately, optimize the patient-physician relationship and clinical care.

## Introduction

Physicians have an important role in building a relationship with patients by not only understanding and treating their medical problems but also by treating the person and their being. This holistic model of clinical care and physician-patient interaction was officially coined as the biopsychosocial model of medicine by George Engel in 1977; it promotes a physician-patient relationship through a physician's understanding of the patient's perspective of their medical illness and by integrating the biological, social, and psychological aspects of their care [1]. His vision of such a clinical model has garnered esteem since his proposition in 1977, as it continues to efface the limiting ideologies of clinical practice of the biomedical and paternalistic models of care by emphasizing humanism and the empowerment of patients. Other models of care like the physician-centered or paternalistic approach to the physician-patient interaction focuses on the physician unilaterally making recommendations to patients without mutual agreement on the direction of clinical care [2]. Part of establishing an appropriate clinical relationship with patients requires the application of bioethical principles of medical ethics, which serve as a paradigm for all medical interactions at a minimum. These principles include patient autonomy, beneficence, non-maleficence, and justice [3]. When these clinical principles are not practiced effectively, patient interaction and clinical rights are not optimized, and clinical errors are more likely to occur, which can be devastating to the patient and their families. This paper is a clinical analysis of a 67-year-old male admitted to a large community-based hospital in South Florida for an acute exacerbation of congestive heart failure, who had sub-optimal clinical care with shortcomings at multiple levels. These limitations include oversight and lack of utilization of the foundational principles of medical ethics, poor inter-team communication, and a general undermining of the role of a hospitalist in the continuity of patient care. This paper aims to review the essential features of clinical ethics as a paradigm for effective communication and the use of the biopsychosocial model of clinical care for optimizing the physician-patient relationship, coordinating care among specialists, and avoiding clinical errors.

## Case presentation

This is a case of a 68-year-old Hispanic male with a medical history of congestive heart failure (CHF) and a reduced ejection fraction of 25% (rEF25%), automatic implantable cardioverter defibrillator, three-vessel disease, resulting in a coronary artery bypass (CABG) arterial hypertension, and end-stage kidney disease (ESKD) (Stage IIIA), who presented to a large community-based hospital in South Florida with severe hypoxic respiratory failure induced by pulmonary edema after the patient's primary care physician reduced his heart failure medication regimen. During his hospitalization, the patient was evaluated by several different medical providers, including being managed by the primary internal medicine team, nephrology, cardiology, and psychiatry.

Clinically, the patient was treated for pulmonary edema as a result of a CHFrEF25% exacerbation. His pro-brain natriuretic peptide (pro-BNP), reflective of volume overload in the atrial chambers, was noted to be over 10,000 at the time of admission. Computed tomography (CT) imaging of the chest/abdomen showed a right pleural effusion with ascites and body wall edema; cardiomegaly was also noted on an anterior-posterior (AP) radiograph of the chest. Routine workup of the patient showed that he had an elevated thyroid-stimulating hormone (TSH), with free T4 values within normal limits, consistent with subclinical hypothyroidism. Despite not requiring treatment as documented by the primary medical team managing the patient’s care, he was issued therapeutic doses of levothyroxine by the consulting nephrology team.

During the patient's hospitalization, he was noted to be aggressive by nursing staff triggering a psychiatric evaluation. The patient was subsequently evaluated by a staff psychiatrist who documented a series of seemingly contradictory labels revolving around a diagnosis of major depression with psychotic tendencies. Such findings included having a flat affect, being inattentive, minimally communicative, disheveled, and anxious. He was also noted to have tangential thoughts, psychotic tendencies, hyperactive at times, and poor eye contact. The list of his behaviors went on to included listlessness, dolefulness, and weariness. 

None of the documented behaviors were observed in the patient by the hospitalist team or his wife of nearly 50 years who was at his bedside during this entire hospitalization. The patient's wife also kept meticulous records since his admission and reported that the psychiatric evaluation of the patient was mostly observational and less than 10 minutes in duration. The clinical diagnosis by psychiatry resulted in the administration of a series of antipsychotic and mood-stabilizing agents.

## Discussion

This case represents a failure of patient care in multiple aspects, many of which most likely may have been prevented by utilizing the biopsychosocial model of clinical care. First, it is important to understand that the patient was admitted to the hospital for an exacerbation of heart failure with hypoxic respiratory failure due to pulmonary edema, making it very difficult for the patient to breathe. Most patients in this circumstance are generally found to have low energy and be minimally responsive. Additionally, the effects of having a New York Heart Failure Class (NYHC) 3 or 4 can be quite depressing for patients. Failing to understand the perspective of the patient led to erroneous labeling of the patient and psychiatric medication that was not justifiable. More so, the essential role of the internist who, in most instances, regularly interacts with the patient frequently, was completely undermined. In theory, the internist could manage heart failure without the need for a cardiologist and renal injuries without the need of a nephrologist. Rather, these specialists are consulted by the internist for the benefit of the patient, given their respective expertise on the matter. However, communication is necessary. Hospitalists review daily labs, perform physical exams, and communicate with the patient and their families with such detail and completeness that interdisciplinary communication between the specialist and the hospitalist would greatly benefit the gross plan of care and outcomes for all parties. In this case, however, discussions with the hospitalist and the nephrologist were not had, and the nephrologist administered doses of thyroxine hormone that was not clinically indicated. Furthermore, hypothyroidism is a condition that is better managed by the hospitalist physician due to detailed knowledge of the most current guidelines for treatment.

Incorporating the bioethical principles of medical ethics into the physician-patient encounter, and utilizing the biopsychosocial model as a clinical practice paradigm, allows for optimized clinical management that is streamlined, effective, professional, efficient, and, hopefully, without clinical error. To begin, the fundamental principles of clinical bioethics include patient autonomy, beneficence, non-maleficence, and justice [4].

Primarily, beneficence in a medical context historically means that physicians “do good” for the patient while offering treatment [4]. In order to do so, physicians must continuously scrutinize their medical decisions and utilize the latest knowledge or evidence available in directing the course of a medical treatment plan. This principle opposes that of non-maleficence, which is the act of doing no harm. In the setting of bioethics, physicians failing to do their due diligence in making such decisions violates the medical ethics principles of non-maleficence. The vagueness of the word “harm” is open to different interpretations but, at a minimum, implies a condition that worsens the patient's baseline status or elicits adverse consequences [4]. In this specific case, the patient with asymptomatic subclinical hypothyroidism was treated with supratherapeutic doses of levothyroxine. Not only was this not medically indicated but it was also potentially lethal to the patient who could have developed cardiac arrhythmias as an adverse reaction to the supratherapeutic levothyroxine. These medical decisions by a consulting physician not only placed the patient’s welfare at risk but they also directly undermined the role of a primary medical internist who would otherwise manage such medical conditions should they need to be treated in the best interest of the patient’s welfare [5]. This risk is especially heightened by the patient’s additional comorbidities of CHFrEF25%, AICD, three-vessel CABG, arterial hypertension, and ESKD. Widely accepted in clinical practice, when iatrogenic errors are made, it is of the greatest importance for the treating physician to inform the patient that such an error was made [6]. Although neglected in this case, the appropriate course of action should have been for the treating physician to apologize for treating the patient inappropriately and discuss possible sequelae that may result from such a treatment. Instead, the treating nephrologist placed orders for the medication to be administered to the patient by the nurse. This was discovered by the hospitalist team when visiting with the patient during morning rounds, and the patient's wife asking why the hospitalist team never explained that he had something wrong with his thyroid. It was later explained that the patient was treated in error and that he was clinically asymptomatic and did not require treatment. Using the biopsychosocial model of care would allow for open dialogue with the patient regarding lab results, the patient's understanding of the illness, and whether or not treatment is indicated. Throughout this process, several iterations of the clinical review of the patient's symptoms, history, and lab results would have occurred. Additionally, the patient would have been more informed regarding their condition, and further workup and discussion with appropriate specialists like an endocrinologist would have been recommended if needed. While some decisions to treat or not treat certain medical conditions are recommended per guidelines, other disciplines of clinical care like psychiatry are more subjective in nature and indications for medical intervention are not so easily elucidated.

The practice of psychiatry requires the mastery of mental health conditions and diagnoses. One of the challenges of the field entails the interpretation of very subjective behaviors, expressions, and attitudes that are heavily influenced by one's persona observed during the physician-patient interaction, culture, and overall health/well-being. Without keen observation and consideration, an incorrect diagnosis of mental illness can result and have very potent effects as was evident in this patient. Certainly, much information regarding a patient's mental health condition can be ascertained during the initial encounter, however, findings of a mental health illness need to be considered in the context of the patient's overall physical health and condition. Evidence of a mental health illness must be confirmed on more than one clinical encounter, which was not the case for this patient. In reviewing the list of observed behaviors documented by the psychiatrist, many of them are contradicting in nature and concerns of inpatient psychiatry would be indicated should the patient have truly demonstrated such a gamut of behaviors during the 10 minutes of observation. Research has shown that patients with multiple comorbidities have a 50% or greater chance of developing depression due to a heightened level of stress secondary to illness(es) [7]. Failing to understand the context of the patient’s source for depression, led the patient to be treated with multiple antipsychotic medications, which were never a part of his medical history. Practicing mindfulness through the biopsychosocial model of care would have allowed for a complete understanding of a patient with difficulty breathing due to a heart failure exacerbation, resulting in a state of lethargy and general listlessness, in combination with possible depression from general poor health and prognosis. A physician must be able to appreciate subtleties in deciphering true depression from a mimicking medical condition and acute delirium vs. psychosis. Additionally, it is of utmost importance for a physician to appreciate gestures and cultural appropriateness. For example, the psychiatrist noted the patient to avoid eye contact. It is unlikely that the patient's perspective was considered in doing those actions. Maybe the patient felt ashamed of his current state of health and the fact that he was generally unkempt, with a general sense of weakness and inability to feed himself. More so, some cultural practices intentionally avoid eye contact as a sign of respect instead of defiance [8]. It is important to note that after a discussion was held with the primary psychiatrist by the patient’s brother who was also a physician and the patient’s outside primary care provider, the patient was medically cleared for discharge home and prior labels attached to the patient by the psychiatrist were reversed.

A lack of communication can result in mistakes that are easily preventable. Interdisciplinary communication is essential to manage the patient when varying specialty consultants are required. In fact, studies demonstrate that hospitals with high interdisciplinary communication yield greater patient satisfaction and staff loyalty [9]. This highlights the need for a physician who is fully aware of the patient's history and needs to utilize effective communication with members of the clinical care team, including nurses, administration, specialists, family, and, most importantly, the patient.

## Conclusions

It is a privilege for physicians to be in a role to care for patients, which is what provides sanctity to the physician-patient relationship. Older models of clinical care, like the biomedical and paternalistic model, fail to consider the integrated nature and humanism in patient care. Utilizing tools like the fundamental principles of medical ethics as a paradigm for a clinical encounter in addition to the biopsychosocial model enhances the patient-physician relationship to reduce harm to the patient and optimize clinical care. While internists/hospitalists are in a prime position to utilize these powerful clinical tools in building an exceptional standard of care, it can be utilized by all clinical providers. Doing so enables the best clinical practice by reducing errors, improving interdisciplinary team communication, and fostering a professionally quintessential patient-physician communication and relationship.
